# Development of a Controlled Injection Method Using Support Templates for the Production of Chemobrionic Materials

**DOI:** 10.1021/acsomega.2c02620

**Published:** 2022-06-23

**Authors:** Bahar Aslanbay Guler, Zeliha Demirel, Esra Imamoglu

**Affiliations:** Department of Bioengineering, Faculty of Engineering, Ege University, Izmir 35040, Turkey

## Abstract

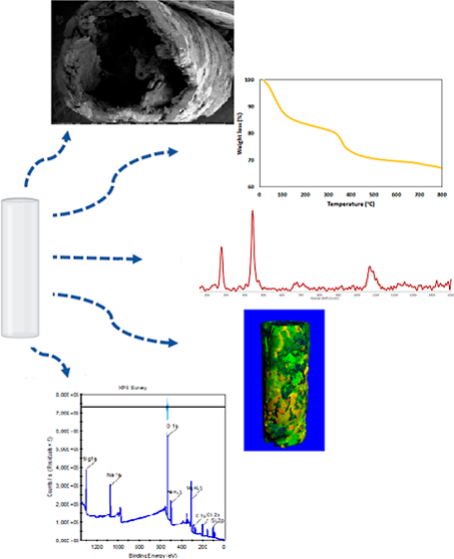

Chemobrionics is a research field about the well-known self-organized inorganic structures. Numerous research works have focused on controlling their growth pattern and characteristic features. In the present study, a controlled injection method is proposed to produce more regular self-assembled chemobrionics compared to the standard direct injection technique. This method involves the injection of a metal salt solution into an agarose support template filled with an anionic solution. The obtained structures were studied by scanning electron microscopy, X-ray microtomography, X-ray photoelectron spectroscopy, Raman spectroscopy, Fourier-transform IR spectroscopy, and thermogravimetric analysis. Despite the complex mechanism and chemistry underlying the self-organization phenomena, the controlled injection method enabled the generation of regular standard chemobrionic structures with high experimental reproducibility. It provided the extraction of tubular structures from the reaction vessel without breakage, thus allowing comprehensive characterization. Furthermore, the morphological, chemical, and thermal features of these structures were highly correlated with the standard chemobrionics obtained in the direct injection method. The proposed controlled injection method holds great promise for understanding and controlling the properties of chemobrionics and related structures.

## Introduction

Over the past few decades, complex self-organizing structures have attracted great interest from scientists due to their role in understanding the molecular origin of life and their promise for a variety of applications. The process of self-organization is the spontaneous arrangement of disordered molecules or ions into well-organized structures under non-equilibrium conditions without external control.^1^ One of the primary examples of these processes is chemobrionics, which is an emerging field of complex systems and nonlinear dynamics. Research related to these systems has focused on the physics, chemistry, fluid dynamics, and growth patterns of biomimetic self-organized structures. There are several types of chemobrionics, including chemical gardens, Liesegang rings, silica-carbonate biomorphs, and so forth. Among them, chemical gardens are one of the most iconic and oldest systems.^2,3^ They are generally developed with a simple procedure; a soluble metal salt seed is placed into an aqueous solution containing reactive anions, and hierarchical tubular structures are formed within seconds to hours. The resulting structures have many characteristic and functional features, such as electrochemical, magnetic, and dynamical properties, depending on their chemical compositions and operating conditions.^3,4^

Recently, there has been great effort made in research focused on chemobrionic production techniques in order to understand the growth mechanism and to develop specific structures. Most common procedures are classified as seed growth, injection growth, membrane growth, and growth in quasi-two-dimensional systems. These methods are highly capable of providing deeper information on the chemical composition and morphology of chemobrionics. Also, they have strong potential for the fabrication of numerous complex biomimetic structures. However, the self-organization mechanism and the irregular growth pattern of chemobrionics lead to the formation of disordered and fragile products that complicate their handling and characterization.^5,6^ In order to overcome this challenge, different production techniques and control strategies have been suggested in the laboratory-scale studies. Some of the specific examples include controlling the wall thickness by bubble guidance, applying oscillatory pressure changes, and using a custom-built liquid exchange unit for chemobrionic production.^7−9^ These approaches have made great contributions to understanding the mechanism behind chemobrionics and manipulating the growth pattern of the structures. Considering that the formation of inorganic membranes are highly specific to several parameters, including chemicals, production techniques, and environmental conditions, many more studies should be undertaken in order to explain and control the growth of chemobrionics based on varying experimental parameters. Furthermore, the common problem with laboratory-scale experiments is that the extraction of chemobrionics from the reaction vessel is highly challenging because of the fragile nature of the products.^10^ Therefore, production techniques should be improved in order to make the extraction, characterization, and application of the structures easier.

**Figure 1:**
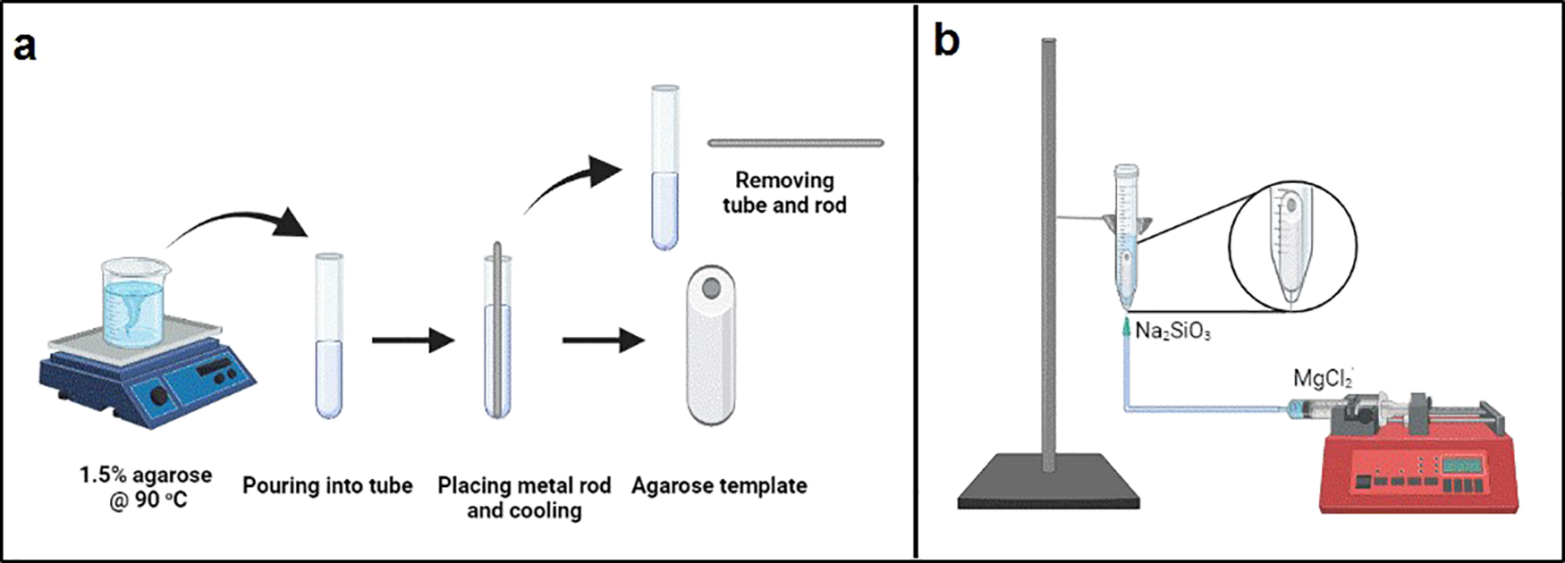
Schematic illustration of the experimental setup. (a) Agarose template production and (b) controlled injection experiment.

In this paper, a controlled injection method is presented to improve the direct injection method in terms of controllability, standardization, and stability. In the standard injection method, a metal salt solution is directly fed into the anionic solution, and chemobrionic structures are formed in varying sizes and random orientations. In this context, the controlled injection method has been proposed to produce regular structures with specific dimensions. The experimental setup of this method consists of an agarose template located in the middle of the reaction vessel which is filled with a silicate solution (Figure 1). The metal salt solution is injected into the template at a constant rate. The injection is carried out in the upward direction for 2 h, and the growth regimes of precipitates are monitored during the experiment. For a more systematic investigation, the direct injection method is also used with the same reactants in order to compare the morphologies of classical chemobrionics and generated structures. The obtained structures are studied by scanning electron microscopy (SEM), X-ray micro-tomography (μ-CT), X-ray photoelectron spectroscopy (XPS), Raman spectroscopy, Fourier-transform IR (FTIR) spectroscopy, and thermogravimetric analysis (TGA).

## Results and Discussion

The present study reports a controlled injection method for the production of regular self-organized structures in a confined geometry by using an agarose support template. For the first step, a hollow agarose template was produced with a simple procedure based on solidifying the agarose in specific dimensions (Figure 1a). Subsequently, chemobrionic production was carried out by the injection of 0.5 M MgCl_2 (aq)_ solution into the agarose support template filled with 2.0 M Na_2_SiO_3 (aq)_ (Figure 1b). For a more systematic investigation, chemobrionic structures were also produced using the standard direct injection method with the same reactants and obtained structures were compared in terms of growth pattern, morphological structure, chemical composition, and thermal features.

### Growth Pattern of Tubular Structures

**Figure 2:**
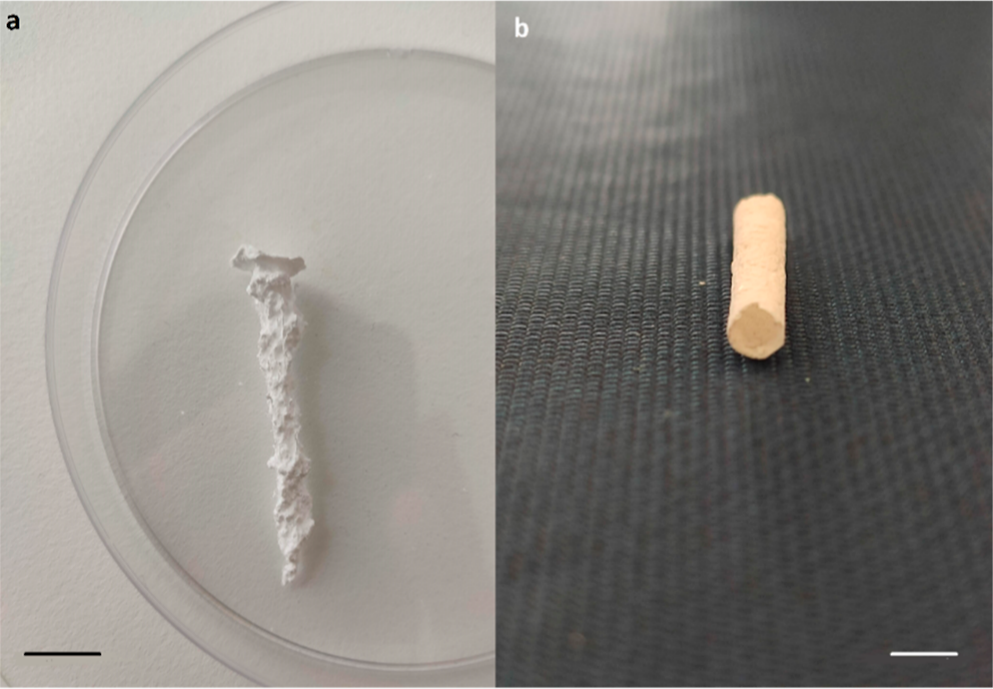
Tubular structures grown by the (a) direct injection experiment (scale bar = 10.0 mm) and (b) controlled injection experiment (scale bar = 3.0 mm).

In the direct injection method, MgCl_2 (aq)_ solution was directly injected into a vessel containing the Na_2_SiO_3 (aq)_ solution at a constant rate of 2 mL h^–1^. When the two solutions mixed, a white precipitate of magnesium silicate began to form, and small tubular pieces split off from the tip of the needle. These pieces raised toward the liquid–air interface and formed a white cloudy cluster at the surface. In other words, any stable membrane structure was not observed for a short time after the injection started. As the flow continued, the precipitate generated in the needle tip grew both vertically and horizontally, producing a wide multi-tubular structure (Figure 2a). It is worth noting that the tubes showed a random growth pattern without any regular organization and thus, different precipitate structures were observed in the repetitive productions. This randomness can be explained by the nature of chemobrionics, which is based on the continuous rupture and reprecipitation of membranes at unspecified locations.^4^ The growth regime and visual morphology of the obtained chemobrionic structures from the direct injection experiment were similar to those of magnesium and calcium salts, especially for the injection techniques.^10,11^

**Figure 3:**
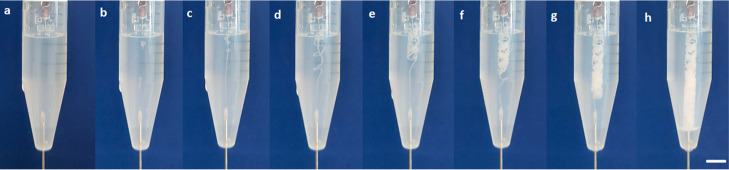
Tubular structure obtained by the controlled injection method. (a) Before injection and after (b) 3 s, (c) 8 s, (d) 15 s, (e) 30 s, (f) 1 min, (g) 2 min, and (h) 5 min of injection (scale bar = 5.0 mm).

The presented controlled injection method offered a higher degree of control over the growth of chemobrionics than the direct injection method. Therefore, the growth pattern of the structures produced in the controlled injection method were photographed at different time intervals to observe the process and control possible fluctuations. On the other hand, the direct injection method is a standard technique used by many researchers, and the growth pattern of chemobrionics in these systems is well known and extensively reported.^11,12^ In the controlled injection experiments, when the salt solution was injected into a silicate solution, several balloon-like precipitates formed, followed by their quick release from the injection point within a few seconds (Figure 3b). Next, a white narrow gel-like tube was generated that reached the top of the template within a few seconds and accumulated at the air–liquid interface creating a precipitate cluster (Figure 3c). The observed growth regime during this initial period was highly consistent with the “jetting” growth reported in the literature,^13,14^ and it shared similarities with the structure in the direct injection experiment. However, the accumulating precipitate at the air–liquid interface was trapped in a confined area restricted by the agarose template, rather than spreading in a disordered regime as in the direct injection method. As the injection continued, the growing jet precipitate showed a slight twisting motion toward the middle of the template due to the mechanically weak nature of the structure. After some seconds, spiral orientation and aggregation were observed along the wall of the template (Figure 3d). This growth pattern lasted for several minutes and then a lamellar morphology which consisted of a tightly packed and folded tubular structure was observed. It was thought that the spiral movement of precipitates was randomly oriented through the actions of osmosis and buoyancy force. The resulting folding pattern was a result of planar zigzag or helix conformations. A similar growth pattern was reported by Knoll et al*.*,^15^ who studied the formation of precipitate structures by the injection of a non-reactive liquid into a thin capillary filled with an immiscible liquid. They observed macroscopic structures including helices, lamellae, and disordered patterns with the changing flow rate and suggested that these morphologies might arise from several factors including small asymmetries in the precipitate membrane, from effects related to corkscrew-like instabilities of fluid jets or from discontinuities in the tangential velocity of fluids. In the present study, this spiral orientation varied in length from 1 mm to a few centimeters, and a more intense tube was observed as the injection continued. Meanwhile, a small thicker precipitate formed at the tip of needle. This precipitate membrane grew vertically and eventually attached to the former intense tubular structure (Figure 3h). In some cases, the formation of fine unstable tubes was observed outside the agarose template. They had grown up from the bottom of the reaction vessel vertically and accumulated as clusters at the top of the air–fluid interface. As further injection was carried out, the continued accumulation resulted in the sinking of clusters to the bottom of the vessel. According to visual observations, external chemobrionic formation did not affect the main structure in terms of growth pattern and morphologic appearance.

The injection process ended after 2 h because from this moment the injected solution passed through the agarose template and slowly accumulated on the top of the tube without any further macroscopic precipitate formation being observed. The tubular structure formed during the entire period did not differ greatly from the precipitate generated in the first minutes, but the initial transparency turned to a more solid appearance at the end of the process. In this method, the tube length and diameter were limited by the dimension of the agarose matrix (Figure 3h). In an additional experiment, two different injection needles with inner diameters of 0.51 and 1.27 mm were used in order to investigate the effect of tip diameter on the growth pattern of tubular precipitates. In the first experiment where the injection was carried out by the thinner needle, the thickness of the initial jet precipitation tube was smaller than the one observed in the second experiment. With continued injection, the appearance of both structures was found to be highly similar. This result was supported by the fact that the structures obtained at the end of both experiments were similar in size and morphology. In brief, the needle diameter had no significant effect on the growth pattern and the morphology of the chemobrionic structure (data not shown).

Visual observations of the resulting dried structures revealed that the controlled injection method provided the production of a single straight tube without any branching. The obtained chemobrionics had a hollow structure with an interior space and a particulate rough exterior surface (Figure 2b). They had a diameter of ∼3.0 mm, which was only slightly smaller than those of the gap inside the agarose template. The height of the tubular structure depended strongly on the size of the agarose template. It was observed that the height of chemobrionics could be increased up to a point by increasing the height of the template. However, further increases in the template height led to random precipitation patterns in the upper regions and thus forming precipitates had a translucent white structure due to the low degree of mineralization. In this case, the stability and rigidity of the structures may be enhanced by the increase of process time because it has been reported that the tubes, which were synthesized in a longer time were more robust and steady.^16^ One of the major obstacles for self-organization structures is the unstable character of tube growth and poor experimental reproducibility.^17^ In this study, the controlled injection method performed with an agarose template gave more reliable and reproducible structures during parallel experiments than the direct injection method and thus, it has the potential to provide new routes to solve the primary challenge of chemobrionics and related structures.

### Characterization of Tubular Structures

One of the most fundamental challenges for inorganic precipitation studies is to extract the tubular structure from the reaction vessel and to prepare them for characterization. These difficulties result from the fragile nature of chemobrionics. Therefore, most studies have conducted bulk analyses such as FTIR, X-ray diffraction, and Raman spectroscopy that do not need an intact structure during analysis. In these methods, the whole structure is collected, mixed, and milled to a powder to obtain a homogenous sample. The results obtained from these analyses offer highly impressive features of complex structures but the development of new procedures is required for a much more detailed characterization of precipitates. In the present study, a developed controlled injection method provided the extraction of tubular constructs from the vessel without fragmentation or breakage and thus allowed comprehensive characterization after the drying step. The produced structures in the controlled injection experiment were more robust and steady than those obtained in the direct injection technique. However, some breakages were still observed, albeit at a lower level when compared to the other methods. The fragile characteristics of produced structures may be associated with the presence of microsized cracks, as reported by Makki and Steinbock.^18^ Here, the micromorphologies and three-dimensional (3D) structures of the unbroken inorganic tubes were studied using SEM and μ-CT. Furthermore, their compositional analysis was examined by XPS, FTIR, and Raman spectroscopy, and the thermal features were determined with TGA.

#### Morphological Analysis

**Figure 4:**
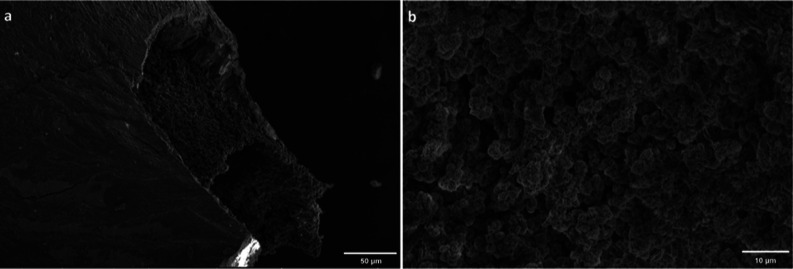
SEM images of magnesium silicate tubes grown by the direct injection method. (a) Exterior surface (scale bar: 50 μm) and (b) interior surface (scale bar: 10 μm).

Chemobrionic structures from direct injection experiments were monitored with SEM and obtained images are presented in Figure 4. The different morphologies between the inner and outer surfaces pointed out the bilayer wall structure and variation in the chemical composition. As shown in Figure 4b, on the internal surface, small crystal aggregates were observed forming different types of morphologies such as needles, spherical pellets, and flower-like microstructures (Figure S1). As reported by several researchers, the microstructure of the tube was highly consistent with precipitates in chemical gardens: the exterior surface of the membrane was smooth whereas the interior was rough.^17,19,22^ Additionally, Sainz-Diaz et al*.*^11^ revealed that the flower-like rosette crystals belong to magnesium oxy/hydroxide microspheres and small spheres indicated the mixture of magnesium silicates.

**Figure 5:**
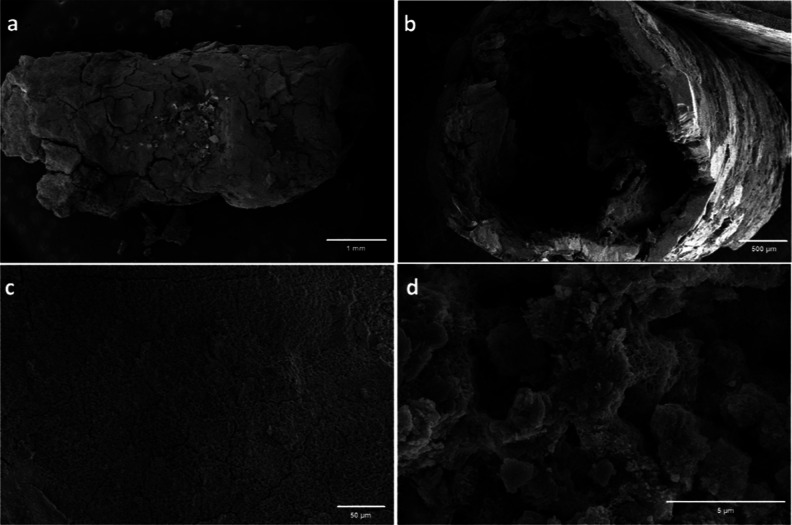
SEM images of magnesium silicate tubes grown by the controlled injection method. (a) General view (scale bar: 1.0 mm), (b) cross-sectional view (scale bar: 500 μm), (c) external surface (scale bar: 50 μm), and (d) internal surface (scale bar: 5 μm).

The SEM images of the structures from the controlled injection experiment showed that the surface texture had an irregular morphology, including heterogeneous cracks and disordered aggregates of different crystals. These microsized cracks were probably responsible for the fragility of the structure, as mentioned above. Analyzed inorganic tubes typically exhibited diameters of ∼3.0 mm, and they had bilayer wall structures as shown in the vertical image (Figure 5b). The microstructure of the wall section showed that the interior of the tube was more particulate, but the exterior surface seemed to be smoother (Figure S2). It is interesting to note that second precipitation structures with diameters of about 10 μm were observed inside of the main tube (Figure 5b). This was probably a result of the spontaneous formation of multitubular structures, which might be derived from the creation of new branches at a junction or the emergence of new tubes directly from the needle tip. The interior surface of the tube was particulate with many small crystals forming spherulites, honeycomb, and rosette structures, similar to the obtained structure in the direct injection experiment (Figures 4b and 5d). As mentioned earlier, these crystals were attributed to the magnesium oxy/hydroxide composition. The obtained SEM results revealed that the tubular structures shared similarities with classical chemobrionics in terms of characteristic surface features, formation of bilayer walls, and magnesium composition.

μ-CT is a powerful technique that uses 3-D high-resolution imaging to observe and quantify the morphometric structure of samples noninvasively. This technique has many advantages including high-resolution, non-destructive analysis, short scanning time, high sensitivity, and the possibility of *in vivo* and *in vitro* monitoring. μ-CT has been successfully applied to imaging different materials in many areas and the key morphological parameters obtained from the analysis are porosity, wall thickness, surface area, and mineralization level.^20^ However, analysis of chemobrionics with μ-CT is challenging because of the fragile nature of structures and according to the literature, there are a limited number of studies that investigated the microstructure of chemical gardens by using this technology.^21^

**Figure 6:**
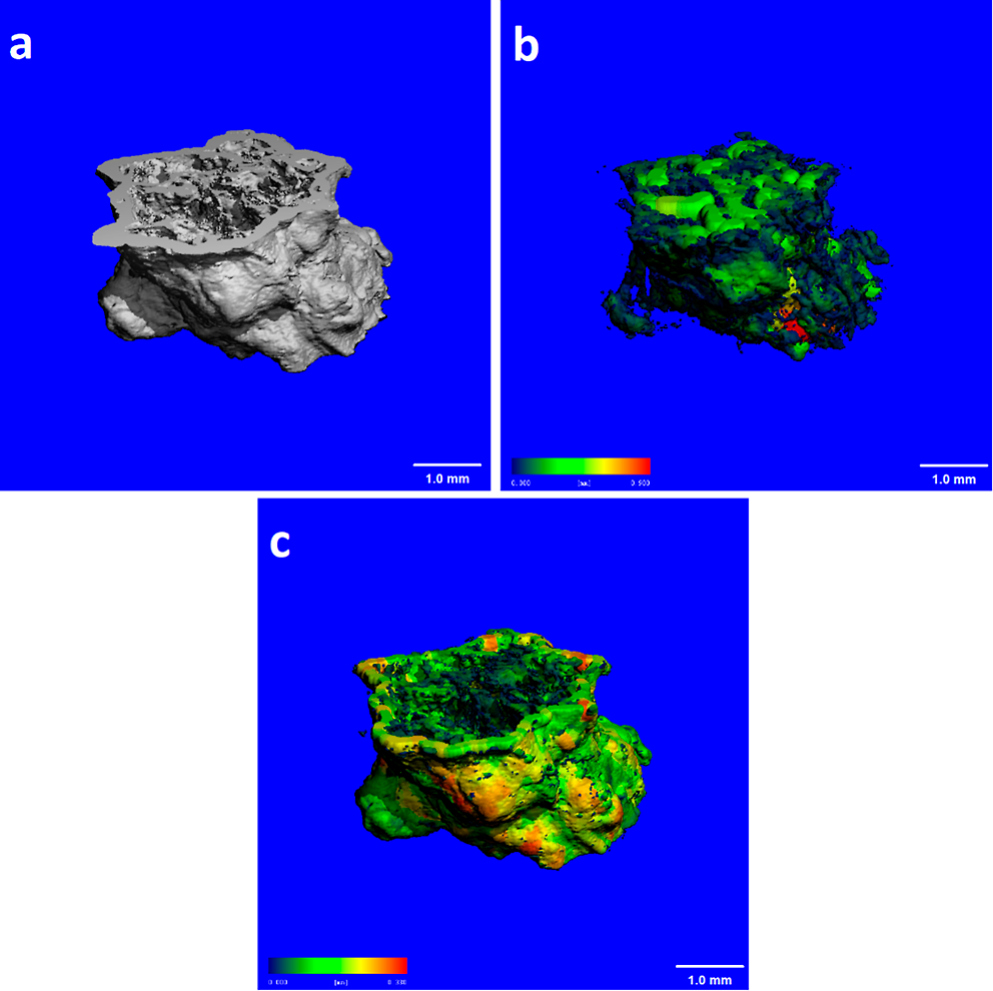
μ-CT analysis images of the chemobrionic structure obtained by the direct injection method (scale bars: 1.0 mm). (a) 3D reconstructed image, (b) pore distribution image, and (c) wall thickness image.

In this study, the produced structures from direct and controlled injection experiments were investigated by μ-CT scanning in order to attain a better understanding of the effect of the developed controlled injection method on the formation mechanism and morphology of chemobrionics. The outputs from μ-CT analysis provided clear and quantitative data on the inner structure, void size (derived from fiber orientations, cracks, and empty spaces), void distribution, wall thickness, and growth pattern of the obtained structures. For the analysis of the structure from the direct injection method, specimens were prepared in lengths of approximately 1.5 mm to reduce the overall processing time. 3D model image, pore/void distribution, and the wall thickness of the structure are illustrated in Figure 6, colored according to their size. Figure 6b shows that a large void volume, which was composed of a non-uniform and a randomly arranged pore structure appeared through the middle of the tube. According to colored distributions, it was observed that the void spaces created large pores, which had sizes up to 800 μm. As a result of analyzing the samples in three replicates, the pore size distribution was determined (Figure S3a-1). The majority of the voids (∼70%) were found in the range of 100–500 μm, while the macroscopic pores had a highly low distribution. The mean pore diameter and percentage porosity values were found to be 305.2 ± 48.5 μm and 58.4 ± 11.2%, respectively. Experimental studies of inorganic precipitate membranes revealed that the pore size distribution of the synthesized chemical gardens is in the meso-pore range from 3 to 100 nm.^4,22^ Such a high pore size obtained in this study is related to the formation of multitubular structure, which was generated simultaneously during the injection process because the growing tubes branched at random locations and they were coupled together. The observed junctions between different tubes created vertical conduits, which may have affected the growth pattern of the membrane by the orientation of the fluid flow across varying gradient regions. These conduits and pore spaces led to the formation of large voids inside the main tubular structure. In addition to the factors arising from the production process, these large voids might be formed during extraction or drying steps. Wall thickness, which is another important output from CT scanning, ranged between 150 and 250 μm with an average value of 165 ± 36 μm for the entire structure (Figures 6c and S3b-1). It should be noted that the standard deviations for the quantitative results were quite large considering the analysis on parallel structures. Variability of the metric parameters was related to the random precipitation pattern and uncontrollable nature of membrane growth. This result indicated that the direct injection method had low standardization and poor experimental reproducibility at micro- and nanometer scales.

**Figure 7:**
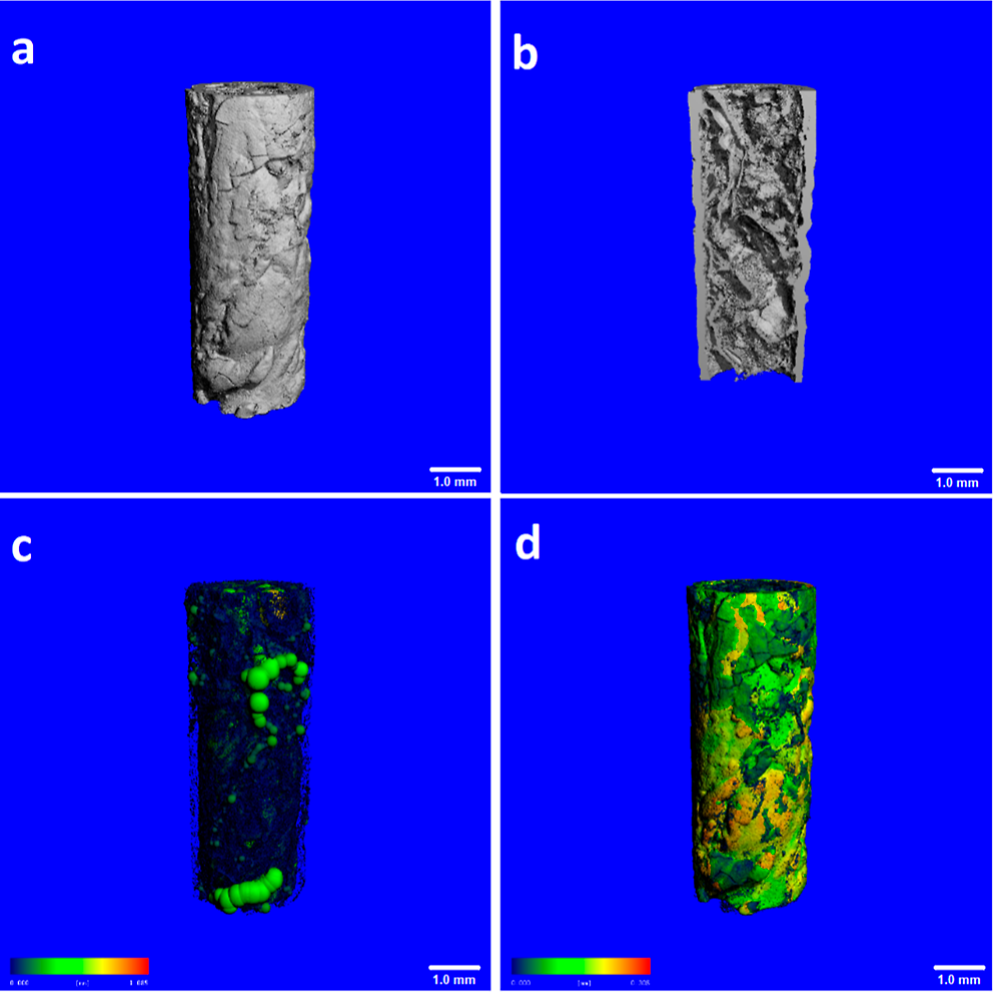
μ-CT analysis images of the chemobrionic structure obtained from the controlled injection method (scale bars: 1.0 mm). (a) 3D reconstructed image, (b) vertical section, (c) pore distribution image, and (d) wall thickness image.

Representative 3D images of the tubular structure from the controlled injection experiment constructed by μ-CT are presented in Figure 7. 3D reconstructive image showed that the wall of the tube possessed capillary cracks, which were consistent with the SEM analysis. The cross section of the investigated structure (Figure 7b) exhibited a remarkably large void volume and non-uniform randomly arranged conduits. These voids and conduits created an interconnected pore network and contributed to the porosity and internal surface area of the structure. When the capillary tubes observed in SEM images (Figure 5b) and the irregular channels in the inner volume of the structure were evaluated together, it can be said that secondary precipitations occurred apart from the main tube. As illustrated in Figure 7c, large voids of various sizes, reaching 1.0 mm in diameter, were randomly distributed inside the structure. The total porosity was calculated to be 79.2 ± 3.8%, with an average pore size of 370.4 ± 10.8 μm. Furthermore, the pore size distribution histogram demonstrated that the structure presented a broader pore size distribution, with a high fraction of macroscopic holes on the order of 100–400 μm, but most of them were smaller than 100 μm (Figure S3b). The wall thickness of the structure was mainly in the range of 150–250 μm and the mean thickness was found to be 145 ± 11 μm. The wall was thickest near the injection point and became thinner with increasing height, resulting in less than 50 μm. μ-CT analysis revealed that the distribution of wall thickness of this tube was more homogenous in comparison with the structure formed by the direct injection method. Roszol and Steinbock^7^ proved that radial wall growth and wall thickness were mainly affected by the diffusion mechanism. In the present study, the use of an agarose template may have provided a controlled diffusion of anionic ions from the silicate solution to the space inside the template, which will be discussed later.

#### Chemical Analysis

The chemical compositions of chemobrionics depend strongly on the reactants, growth method used, and physical conditions. A detailed chemical analysis allows for the investigation of the growth pattern and diffusion mechanism of the produced structures.^4^ Besides, it provides information about the potential existence of unexpected constituents, which may be resulting from the adsorption of atmospheric CO_2_ or oxidation of the reactants. Here, the chemical compositions of the produced structures were characterized by XPS, Raman spectroscopy, and FTIR. The XPS full survey spectra revealed that the surface of both structures consisted of Mg, Si, Na, Cl, O, and C (Figures S4 and S5). In the high-resolution scans of chemobrionic structures from the direct injection experiment, Mg demonstrated only one peak at a binding energy of 1303.51 eV that corresponded to the Mg 1s of the Mg–O.^23^ As shown in Figure S4c, two peaks located in the Si 2p spectrum of the tube at 103.66 and 104.89 eV were attributed to SiO_2_ and SiOH, respectively.^24,25^ The tubular structure from the controlled injection experiment showed a similar Mg 1s spectrum, which had a strong peak at 1303.65 eV, corresponding to Mg–O (Figure S5b). The high-resolution spectrum of Si 2p possessed two peaks at 103.2 and 105.1 eV corresponding to SiO_2_ and Si–OH bonds, respectively (Figure S5c).^26,27^ Chemical composition analysis of these structures revealed that the characteristic peak corresponding to magnesium silicate components could not be observed in XPS spectra, which was explained by the fact that the external surface was formed mainly with magnesium oxide/hydroxide, while the magnesium silicate composition might be predominant in the internal part. Also, the molar ratio of magnesium and silicate (Mg/Si) was found to be 1:1 for both samples. These findings specified that the surface chemical composition of tubular structures from both experiments possessed similar components.

According to Raman spectra of the structures, both tubes had bands at 280 and 444 cm^–1^, which were assigned to lattice modes of brucite Mg(OH)_2_ (Figure S6).^28,29^ However, these two peaks of the chemobrionics from the controlled injection experiment were more intense and broader than those obtained in the direct injection method. This could be related to higher amounts of magnesium involved in the formation of the structure, although the same amounts of magnesium chloride were initially injected for both experiments. The Raman spectrum of the structure of the controlled injection method showed a very weak band at 680 cm^–1^, which corresponded to structures of hydroxylated magnesium silicate. According to the literature, Raman spectra of the silicates contain several peaks at three frequency regions, including low frequencies of 400–700 cm^–1^, intermediate frequencies of 700–800 cm^–1^, and high frequencies of 800–1200 cm^–1^. In the intermediate frequency region, both structures had broader weak bands at 710 cm^–1^ resulting from Si–O–Si symmetrical binding. In addition, two peaks at 1070 and 1100 cm^–1^, which were observed in controlled injection and direct injection experiments, respectively, were assigned to antisymmetric Si–O–Si stretching.^30,31^ Increased intensity and the shift to higher wavenumbers of the band derived from Si–O–Si bonding in the direct injection experiment compared to the controlled injection method suggested a higher polymerization degree of silica content. Besides the common bands, chemobrionic structure synthesized in the direct injection experiment had a weak band at 230 cm^–1^, which was assigned to the lattice vibration of crystalline magnesium chloride. The comparison of Raman spectra of the two structures indicated that they had a similar chemical composition in terms of molecular orientation of Mg and Si elements, but the Mg content was higher in the structure obtained from the controlled injection method, while the silica was more dominant in the direct injection method. This was probably a result of the amount of reactants that participated in the precipitation reaction. In the controlled injection method, the amount of silica solution is about 0.5 mL due to the confined area forming by the agarose template. On the other hand, all 10 mL of silicate solution was ready to react with MgCl_2_ in the direct injection experiment. As a result, the controlled injection experiment produced magnesium-rich chemobrionics, while the direct injection experiment generated silicate-rich materials. Raman spectra of the structures were highly consistent with the FTIR results, which showed that both structures exhibited almost the same vibrational bands at 3700, 1640, 1400, 997, and 430 cm^–1^ (Figure S7). These bands were assigned to the stretching of hydroxyl group (3700 and 1640 cm^–1^), antisymmetric stretching of CO_3_^2–^ group (1400 cm^–1^), stretching of Si–O (997 cm^–1^), and bending vibration of Mg–O (430 cm^–1^).^32^

FTIR analysis showed that the chemobrionic structure obtained from the controlled injection experiment did not exhibit any bands for the agarose, which has main peaks at 3400, 1070, 930, and 890 cm^–1^ according to the literature.^33^ Agarose is a natural polysaccharide that has a broad range of pore sizes (1–900 nm) in its gel form. It is commonly used as a gelling agent for the diffusion of small molecules and ions due to its neutral structure and interconnected pore distribution^34,35^ In the controlled injection method, this ionic mobility may have resulted in a more controlled radial growth of the chemobrionic wall structure. However, it did not show any effect on the growth pattern and chemical composition of the chemobrionic. Although agarose gel is considered as an inert background, there have been some studies that reported the concentration and pH of agarose gel may change the precipitation pattern because of the varying pore sizes and internal surface areas of the gel.^36^ Therefore, the effects of concentration, pH, and thickness of agarose template should be evaluated for further research.

#### Thermal Analysis

Thermal features of tubular structures were evaluated by the TG analysis, and the obtained curves are presented in Figure S8. For both structures, there were two main steps of weight loss, which occurred between 30–200 and 250–500 °C. The first step was related to the physically bound water adsorbed on the surfaces of the tubes resulting in a mass loss of ∼16% of the total weight. The second weight loss between 250 and 500 °C might be attributed to the dehydroxylation of Mg–OH and Si–OH groups.^37,38^ TGA results indicated that the weight change of structures from direct and controlled injection experiments showed similar patterns with total mass losses of 31.54 ± 1.68 and 32.78 ± 2.14%, respectively. According to the literature, the results obtained from TGA can provide initial clues regarding the chemical composition of samples with the combinational application of some other analysis.^39^ In this study, similar TGA curves implied that the chemical content of both structures was also similar in terms of main components.

## Conclusions

The objective of this study was to gain more control over the production of chemobrionics, to enhance their standardization and reproducibility, and to obtain the whole structure from a reaction vessel without any disruption for detailed characterization. The presented novel method, namely, controlled injection, showed that the obtained structure of the controlled injection method was a single, straight hollow tube with a diameter of ∼3.0 mm and it had a bilayer wall structure with a smoother external surface and particulate interior surface. When the novel controlled injection method was compared with the standard direct injection method, it was recorded that the microstructure, chemical composition, and thermal features of both structures shared similarities in terms of surface textures, presence of bilayer wall, main components, and thermal degradation. However, the chemobrionics of the controlled injection experiment was more porous than those obtained in the direct injection technique. Also, the Mg content was higher in the former structure, while the silica was more dominant in the direct injection method.

In conclusion, the developed method provides regular standard macrostructures having specific characteristics consistent with the standard chemobrionics of injection methods. It produces reliable and reproducible structures during parallel experiments and thus, it may provide new routes to solve the challenges of chemobrionics and related structures. Moreover, chemobrionic structures synthesized by the controlled injection method hold great promise for several applications including materials science, sensor technology, catalysis, and filtration systems. Future studies will focus on gaining an understanding of the mechanism underlying the growth pattern of the structure, evaluation of the effect of agarose on precipitation, application of these methods for different reactants, and investigation of the impacts of operation conditions such as flow rate, temperature, and pH of reactants on the chemobrionics.

## Experimental Section

### Materials

Magnesium chloride (MgCl_2_) and sodium silicate (Na_2_SiO_3_) were acquired from Alfa Aesar and Sigma-Aldrich, respectively. All chemicals were of analytical grade and their aqueous solutions were prepared with deionized water. Agarose powder used for template production was obtained from Bio Basic Inc.

### Methods

#### Preparation of Solutions

The preparations of 0.5 M MgCl_2_ (ρ = 1.056 ± 0.002 g/cm^3^) and 2.0 M Na_2_SiO_3_ (ρ = 1.2234 ± 0.001 g/cm^3^) solutions were done by dissolving powder chemicals in distilled water, and they were degassed to provide complete dissolution and avoid the generation of bubbles. The pH of the reactant solutions measured with a benchtop pH meter (HI 2211, Hanna Instruments, USA) and they were adjusted by using 1.0 M HCl and 1.0 M NaOH to 2.5 and 13.5 for the metal and silicate solutions, respectively.

#### Preparation of the Agarose Template

Agarose template was prepared by dissolving 1.5% agarose in 10 mL distilled water with continuous stirring at 200 rpm and the solution was slowly heated to 80–90 °C. After the solution became clear, 2 mL of warm gel was transferred to a clear cylindrical tube having a height of 50 mm and diameter of 7 mm. Then, a metal rod (2.5 mm × 50 mm diameter × height) was placed immediately through the center of a gel to make a hollow cylindrical template. The gel in the tube was allowed to cool and mature at room temperature for at least 30 min. Finally, the gel was pushed out from the tube, a metal rod was gently removed from the material and a transparent cylindrical agarose template with a capillary gap in the middle was obtained. The template was cut into standard pieces (30 mm in length and 7.0 mm in diameter) (Figure 1a) and stored at 4 °C until the experiment.

#### Controlled Injection Experiments

The chemobrionic synthesis process was performed with a vertical flow injection technique, as illustrated in Figure 1b. Throughout the experiments, 4 mL of 0.5 M MgCl_2_ solution was injected from the center of agarose template placed in a 15 mL centrifuge tube (Isolab, Wertheim, Germany) containing 10 mL of 2.0 M sodium silicate solution. The injection was carried out by a syringe pump (New Era-100, USA) at a constant volumetric flow rate of 2 mL h^–1^ through a needle (inner diameter = 0.51 mm; outer diameter = 0.82 mm) placed in the center of the bottom of the centrifuge tube. Experiments were performed at 25 ± 2 °C for 2 h and at the end of the process, the agarose template and its precipitate were removed from the silicate solution. They were washed three times with distilled water to clear the surface of the template and then stored at +4 °C overnight to prevent agarose dissolution. After 12 h, the agarose template was cut with a sharp scalpel and the precipitates were gently extracted from the support template, rinsed in water three times, and allowed to dry at room temperature. The resulting structures were stored in a desiccator under a relatively dry atmosphere until the characterization analysis.

#### Direct Injection Experiments

Besides the controlled injection method, direct injection experiment was performed in order to investigate the growth pattern of Mg-silicate chemobrionics without using a support (agarose) template. In this context, 4 mL of 0.5 M MgCl_2_ solution was injected *via* a syringe pump at a flow rate of 2 mL h^–1^ into 10 mL of a 2.0 M sodium silicate solution. The structures were collected after 2 h of growth, carefully washed, and dried for 24 h at room temperature. Each experiment was performed at room temperature, photographed by using a digital camera at different time intervals and reproduced at least three times.

#### Analytical Techniques

For the characterization of the obtained structures, SEM, μ-CT, XPS, Raman spectroscopy, and TGA were employed in order to reveal the microstructure, the local chemical composition, and the entire 3D structure of the precipitates. SEM analysis were performed on a Thermo Scientific Apreo S instrument (ThermoFisher Scientific, USA). Prior to analysis, chemobrionics were placed on an aluminum stub using double sided sticky carbon discs and gold sputter-coating was performed. SEM images were collected from the system operating at low vacuum and room temperature. μ-CT scans were done using a Scanco Medical μCT50 (Switzerland) device with a source voltage of 70 kVp, a source current of 114 μA intensity, an integration time of 300 ms, and 5 μm voxel size (3D pixel). Slice data was obtained and constructed into 2D images by the system. The images of 2D slices were segmented using a constant threshold across the specimens and analyzed using the evolution program (Scanco Medical, Switzerland) to render 3D images and obtain a quantitative result about the pore size distribution and wall thickness. XPS measurements were done using a Thermo Scientific K-Alpha (USA) spectrometer equipped with a monochromatic Al-Kα source (1486.68 eV) containing a multi-channel detector. Measurements were carried out in the constant pass energy mode at 50 eV, using a 400 μm spot size. The chemical speciation of the elements were attained from the high-resolution spectra of the core-level for Mg 1s, Si 2p, C 1s, O 1s, Na 1s, and Cl 2p. Raman spectroscopy analyses were operated with an InVia Raman microscope (Renishaw Plc, UK) connected with a monochromatic 532 nm laser and 2400 l/mm grating. The presented scan was an average of three accumulations acquired at 100% power. FTIR analysis was performed using a Spectrum Two spectrometer (Perkin Elmer, USA) at room temperature in the range of 400–4000 cm^–1^. Lastly, thermogravimetric tests were performed using TA Instruments SDT Q600 analyzer equipment (New Castle, USA). The samples were heated from 25 to 800 °C at a heating rate of 10 °C min^–1^ under a nitrogen atmosphere.

All the experimental analyses were done in triplicates and presented in figures with the average values.

## Supporting Information Available

SEM images, pore size and wall thickness distribution graphs, XPS spectra, Raman spectra, FTIR spectra, and TGA curves of chemobrionic structures (PDF)
