# Supplementary material for: Development of a Controlled Injection Method Using Support Templates for the Production of Chemobrionic Materials
Source: ACS Omega. 2022 Jun 23;7(27):23910–8. doi: 10.1021/acsomega.2c02620 (PMC9281318; doi:10.1021/acsomega.2c02620)
Supplement: Supplementary file 1 — ao2c02620_si_001.pdf [file ao2c02620_si_001.pdf]

Development of a Controlled Injection Method Using Support Templates for the Production of Chemobrionic Materials

Bahar Aslanbay Guler[¶], *Zeliha Demirel*[¶], *Esra Imamoglu*[¶,*]

[¶]Department of Bioengineering, Faculty of Engineering, Ege University, Izmir, 35040, TURKEY

* Corresponding Author: Esra Imamoglu, esra.imamoglu@ege.edu.tr

SUPPLEMENTARY INFORMATION

Contents:

Figure S1. SEM images of the magnesium silicate tubes grown by the direct injection method, a. exterior surface (scale bar: 25 μm), b. interior surface (scale bar: 5 μm).

Figure S2. SEM images of the magnesium silicate tubes grown by the controlled injection method, a. exterior surface (scale bar: 30 μm), b. interior surface (scale bar: 3 μm).

Figure S3. Pore size and wall thickness distribution graphs obtained by the μ-CT data; a. pore size distribution, b. wall thickness distribution; 1. direct injection method, 2. controlled injection method

Figure S4. XPS spectra of the chemobrionic structure grown by the direct injection method a. full spectrum, b. Mg 1s, c. Si 2p

Figure S5. XPS spectra of the chemobrionic structure grown by the controlled injection method a. full spectrum, b. Mg 1s, c. Si 2p

Figure S6. Raman spectra of chemobrionic structure grown by the direct injection method (blue), controlled injection method (yellow)

Figure S7. FTIR spectra of chemobrionic structure grown by the direct injection method (blue), controlled injection method (yellow)

Figure S8. TGA curves of chemobrionic structures grown by the direct injection method (blue), controlled injection method (yellow)

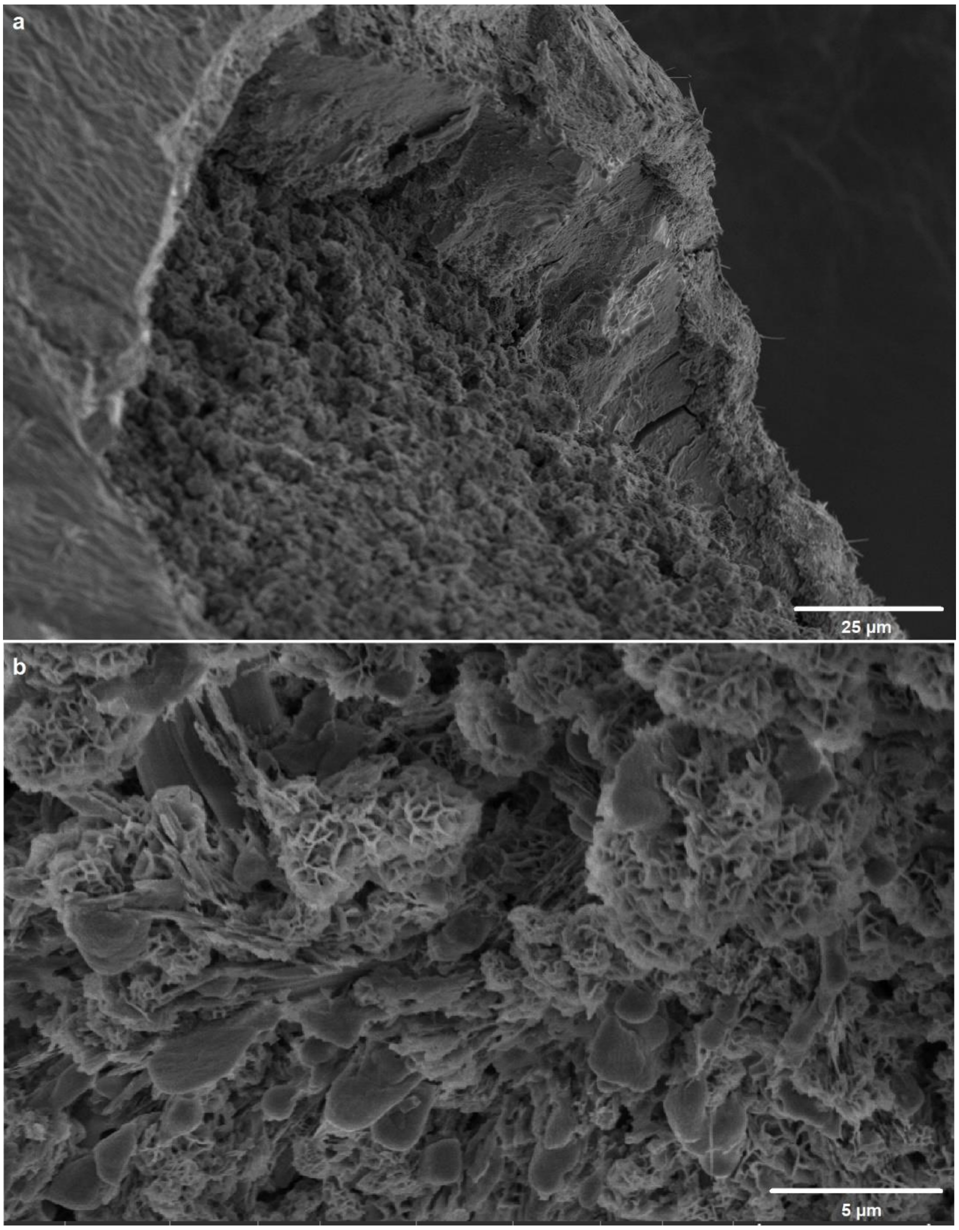

Figure S1. SEM images of the magnesium silicate tubes grown by the direct injection method, a. exterior surface (scale bar: 25 μm), b. interior surface (scale bar: 5 μm).

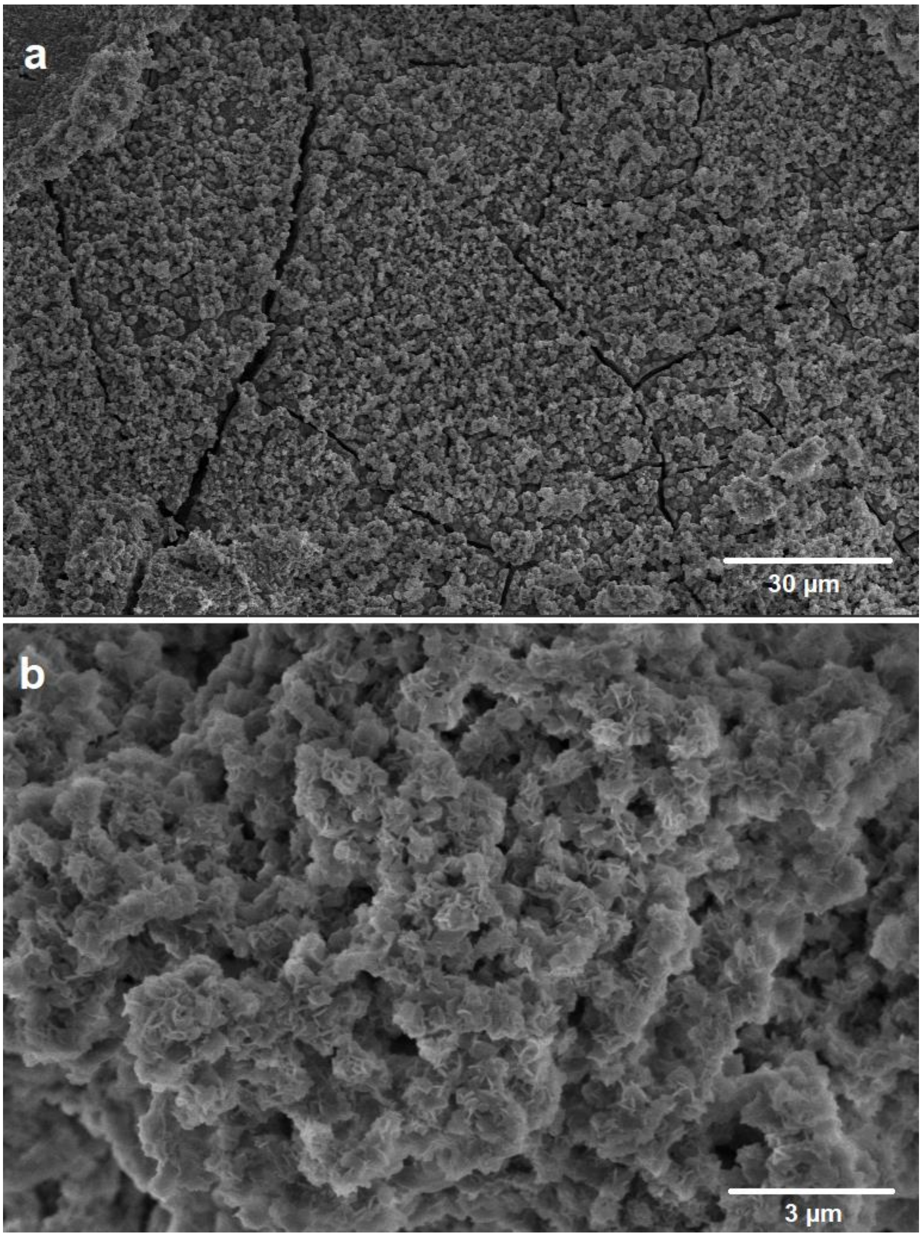

Figure S2. SEM images of the magnesium silicate tubes grown by the controlled injection method, a. exterior surface (scale bar: 30 µm), b. interior surface (scale bar: 3 µm).

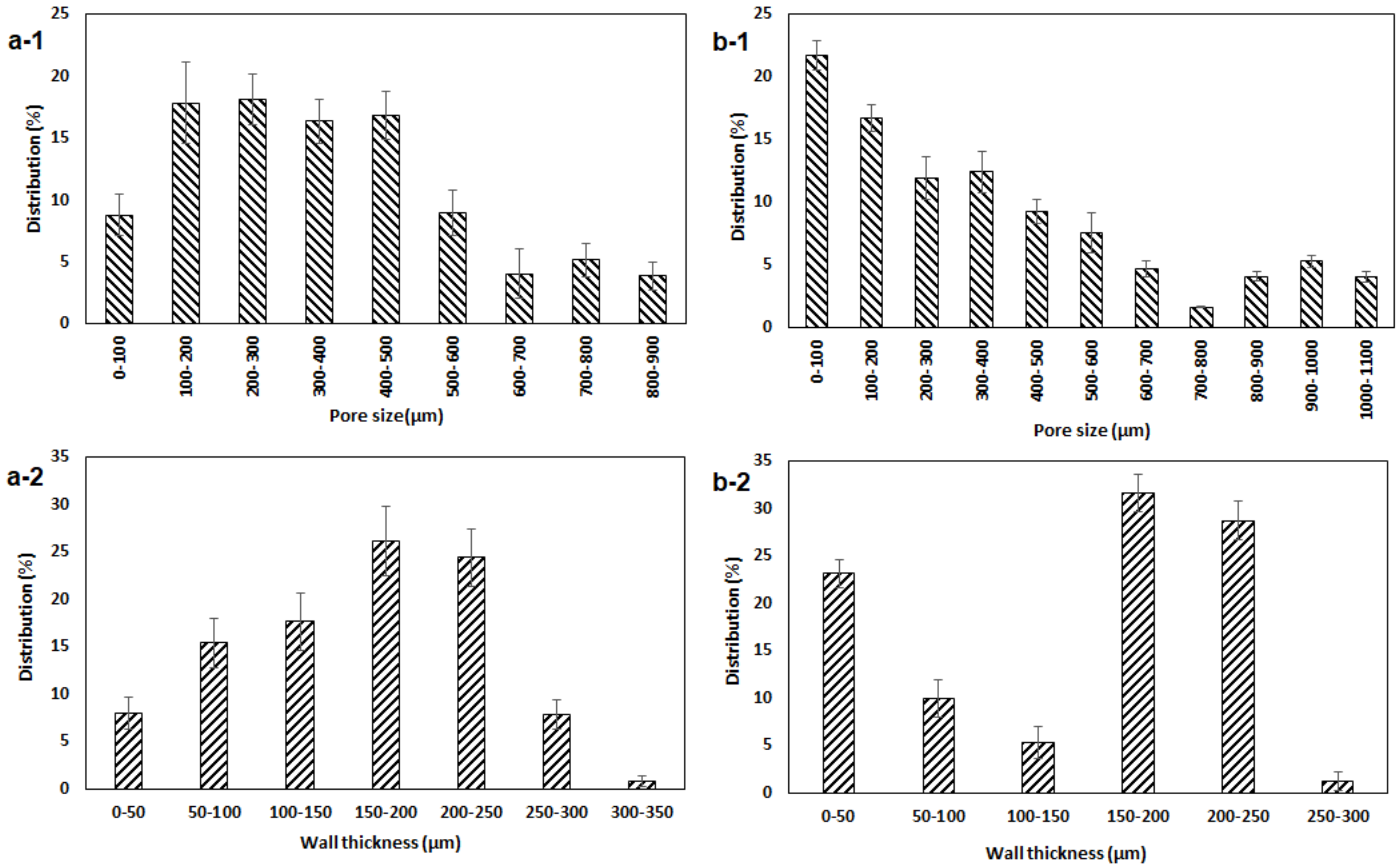

Figure S3. Pore size and wall thickness distribution graphs obtained by the µ-CT data; a. pore size distribution, b. wall thickness distribution; 1. direct injection method, 2. controlled injection method

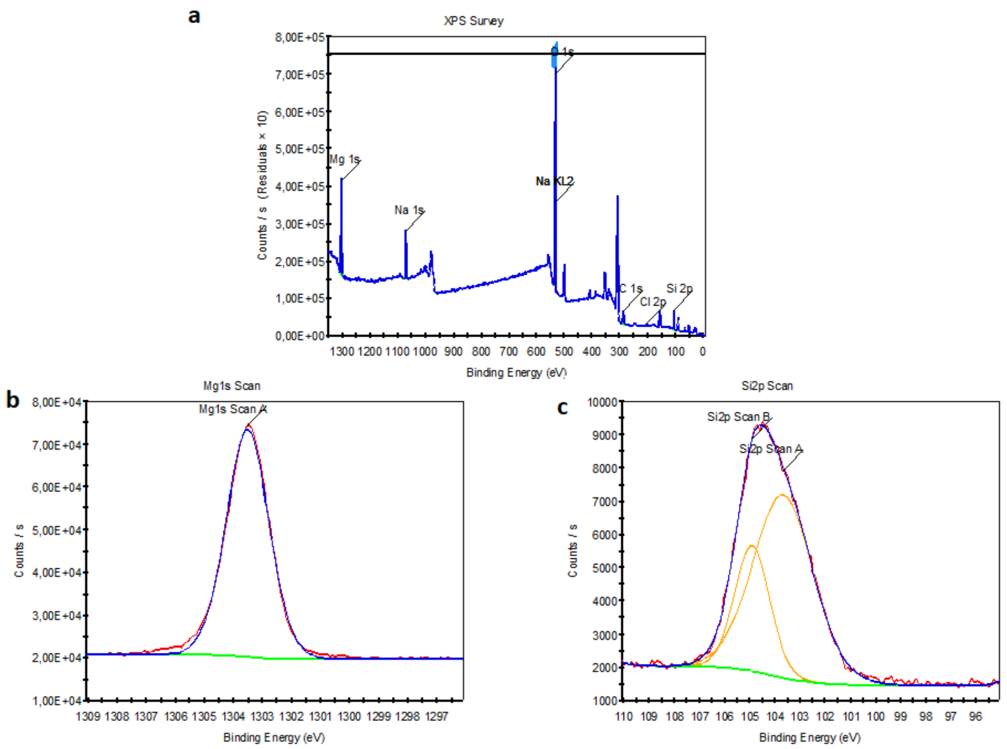

Figure S4. XPS spectra of the chemobrionic structure grown by the direct injection method a. full spectrum, b. Mg 1s, c. Si 2p

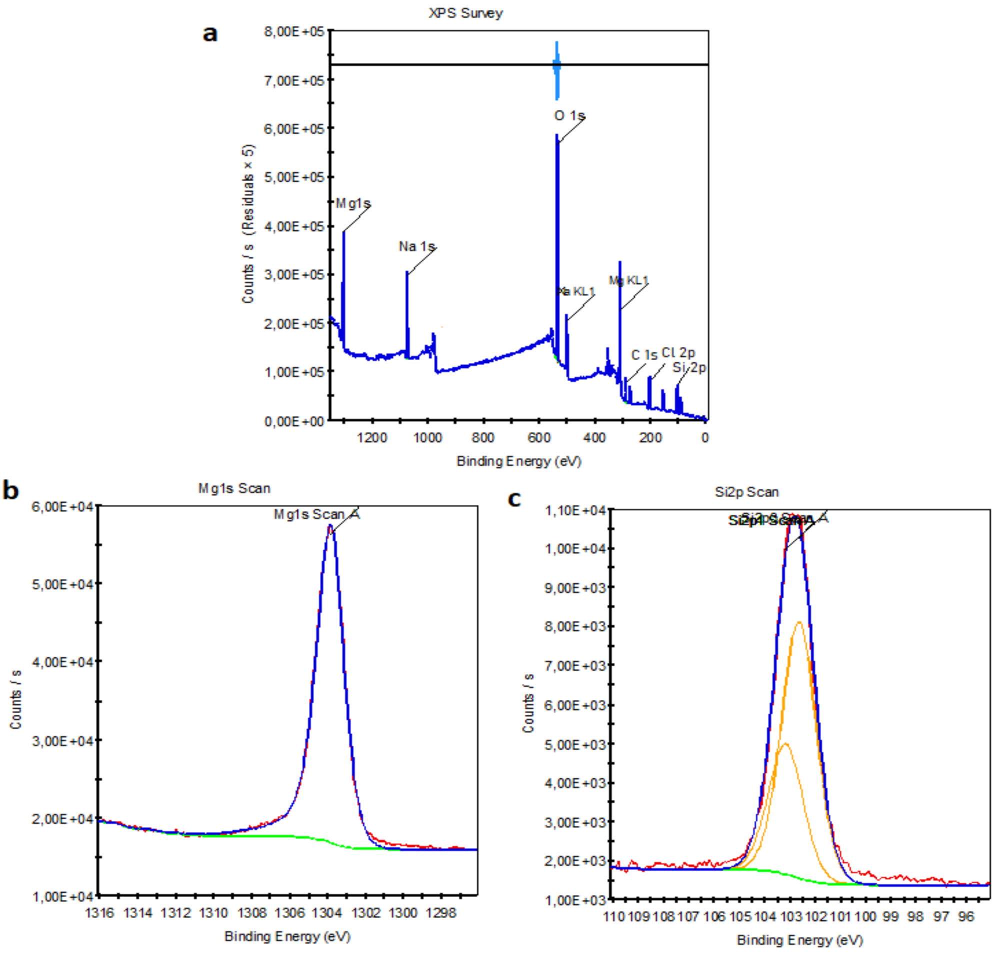

Figure S5. XPS spectra of the chemobrionic structure grown by the controlled injection method a. full spectrum, b. Mg 1s, c. Si 2p

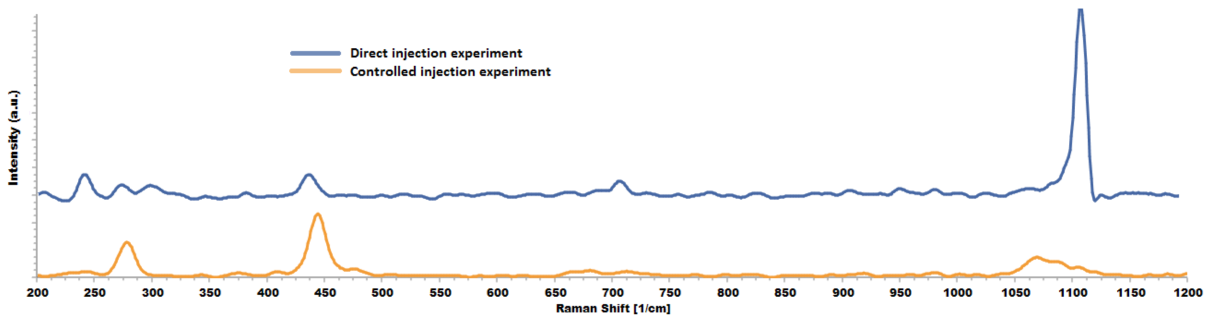

Figure S6. Raman spectra of chemobrionic structure grown by the direct injection method (blue), controlled injection method (yellow)

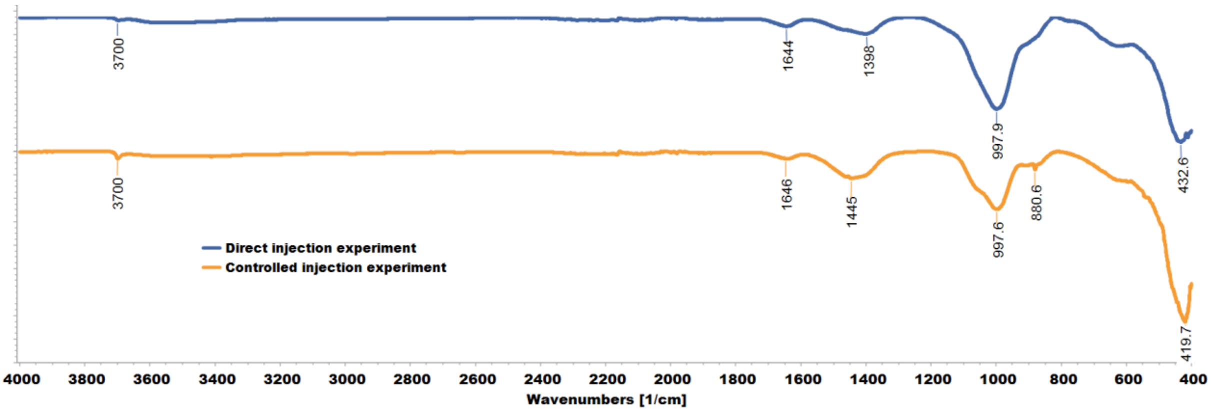

Figure S7. FTIR spectra of chemobrionic structure grown by the direct injection method (blue), controlled injection method (yellow)

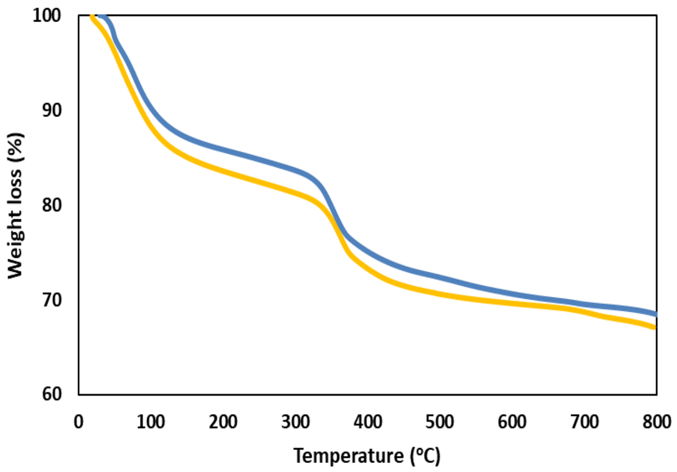

Figure S8. TGA curves of chemobrionic structures grown by the direct injection method (blue), controlled injection method (yellow)
